# *Loktanella* spp. Gb03 as an algicidal bacterium, isolated from the culture of *Dinoflagellate Gambierdiscus belizeanus*

**DOI:** 10.14202/vetworld.2016.142-146

**Published:** 2016-02-12

**Authors:** Anmar Hameed Bloh, Gires Usup, Asmat Ahmad

**Affiliations:** 1 School of Bioscience and Biotechnology, Faculty of Science and Technology, University Kebangsaan Malaysia, 43600 Bangi, Selangor, Malaysia; 2 School of Environmental and Natural Resources Sciences, Faculty of Science and Technology, University Kebangsaan Malaysia, 43600 Bangi, Selangor, Malaysia

**Keywords:** algicidal activity, *Gambierdiscus belizeanus*, *Loktanella* sp. Gb-03, optimization growth

## Abstract

**Aim:**

Bacteria associated with harmful algal blooms can play a crucial role in regulating algal blooms in the environment. This study aimed at isolating and identifying algicidal bacteria in *Dinoflagellate* culture and to determine the optimum growth requirement of the algicidal bacteria, *Loktanella* sp. Gb-03.

**Materials and Methods:**

The *Dinoflagellate* culture used in this study was supplied by Professor Gires Usup's Laboratory, School of Environmental and Natural Resources Sciences, Faculty of Science and Technology, University Kebangsaan Malaysia, Malaysia. The culture was used for the isolation of *Loktanella* sp., using biochemical tests, API 20 ONE kits. The fatty acid content of the isolates and the algicidal activity were further evaluated, and the phenotype was determined through the phylogenetic tree.

**Results:**

Gram-negative, non-motile, non-spore-forming, short rod-shaped, aerobic bacteria (Gb01, Gb02, Gb03, Gb04, Gb05, and Gb06) were isolated from the *Dinoflagellate* culture. The colonies were pink in color, convex with a smooth surface and entire edge. The optimum growth temperature for the *Loktanella* sp. Gb03 isolate was determined to be 30°C, in 1% of NaCl and pH7. Phylogenetic analysis based on 16S rRNA gene sequences showed that the bacterium belonged to the genus *Loktanella* of the class Alphaproteobacteria and formed a tight cluster with the type strain of *Loktanella pyoseonensis* (97.0% sequence similarity).

**Conclusion:**

On the basis of phenotypic, phylogenetic data and genetic distinctiveness, strain Gb-03, were placed in the genus *Loktanella* as the type strain of species. Moreover, it has algicidal activity against seven toxic *Dinoflagellate*. The algicidal property of the isolated *Loktanella* is vital, especially where biological control is needed to mitigate algal bloom or targeted *Dinoflagellates*.

## Introduction

The widespread of poisonous cyanobacterial blooms in rivers, lakes and reservoirs globally has become an increasing health problem. These blooms are also responsible for livestock death and concurrently posing great public health challenge, in addition, to their havoc on the aquatic ecosystems [1-3]. Several bacteria isolated in association with various harmful algal bloom (HAB) species, including dinoflagellates and raphidophytes, have been shown to have algicidal activity [4-8]. Bacteria associated with HABs can play an important role in regulating algal blooms in the environment [9-11]. Laboratory studies had evaluated bacteria or bacteria-derived algicidal compounds to develop a short-term solution for controlling HABs growth [12-17]. These studies showed that algicidal bacteria can exhibit a range of specificity and may use mechanisms to mediate algicidal effects on targeted *Dinoflagellate* species. Many studies have found algicidal bacteria which have activity on a specific genus or species of *Dinoflagellate* [18-20] while others studies showed algicidal bacteria has activity against range of algal classes [21-23].

On the other hand, bacteria with algicidal activities such as those from the genus *Loktanella* has been described [24], to originally contained three species, namely *Loktanella salsilacus*, *Loktanella fryxellensis*, and *Loktanella vestfoldensis*. Subsequently, six other species from this genus were described, these includes *Loktanella* *hongkongensis* [25,26], *Loktanella agnita* and *Loktanella rosea* [27], *Loktanella koreensis* [28], *Loktanella maricola* [29] and *Loktanella atrilutea* [30] were described.

To the best of our knowledge, there is no study investigating marine algicidal bacteria against toxic *Dinoflagellates* in Malaysia water. We hypothesized that bacteria isolates from Malaysian water possess strong and powerful algicidal activity against wide range of *Dinoflagellates* species. Attempts to isolate algicidal bacteria from Malaysian water could reveal the potentials of Malaysian marine as a good source of natural algicides, and they may be of great importance in term of HAB control.

Hence, this study aimed at isolating marine algicidal bacteria from *Dinoflagellates* culture and to optimize their growth requirement. Here we report the taxonomic characterization of a *Loktanella-*like bacterial, Gb-03, which was isolated from *Dinoflagellate* culture.

## Materials and Methods

### Ethical approval

There is no need of ethical approval for such type of study.

### Source of *Dinoflagellate* culture

The *Dinoflagellate* culture used in the study contain *Dinoflagellate* species such as *Coolia malaynesis*, *Alxandrium* sp., *Alxandrium leei, Alxandrium affine*, *Alxandrium tamiyavanichi, Alxandrium tamarense, Gambierdiscus belizeanus*, and *Ostreopsis*. The culture was kindly supplied by Professor Gires Usup, School of Environmental and Natural Resources Sciences, Faculty of Science and Technology, University Kebangsaan Malaysia, 43600 (UKM) Bangi, Malaysia. The culture was continuously cultivated in ES-DK medium [31].

### Isolation

*Dinoflagellate* culture from samples in UKM marine laborator were used for the isolation, and the algicidal activity of the most consistent isolate of the six isolates was further investigated. For isolation, standard dilution plating technique at 25°C on marine agar 2216 (MA;Difco) was used. The resultant suspension was serially diluted in 10-fold steps by the addition of 1 ml of the previous dilution to 9 ml saline, 0.1 ml aliquots from each diluted suspension were spread on MA 2216 (Difco) plates. The plates were then incubated under aerobic conditions at 25°C for 5 days. The morphological, physiological and biochemical characteristics of isolates were investigated using routine cultivation on MA at 30°C for 36-48 h. Isolates were maintained on MA at 4°C for short-term preservation and as a glycerol suspension (20%, w/v in distilled water) at −80°C for long-term preservation.

### Characterization

Growth at various NaCl concentrations was investigated using nutrient broth (NB) 2216 (NB; Difco). Growth at various temperatures (4-45°C) was measured on MB. Growth on NB (Difco), trypticase soy broth (TSB; Difco), MacConkey broth (Difco) and URE broth base were tested.

Colony morphology and pigmentation were determined using a culture grown at 30°C for 36-48 h. Cell morphology was observed under light microscopy (E600; Nikon). Motility was assessed on a semi-solid agar tube containing marine broth (Difco) supplemented with 0.4% agar. Cells were inoculated by stabbing with a straight needle and the tube was incubated at 25°C for 5 days. Gram-staining was performed using a Gram-stain methods. Growth under anaerobic conditions was determined after incubation in a forma anaerobic chamber on MA plate for 48 h at 30°C. Growth in the absence of NaCl was investigated using NB (Difco). Oxidase and catalase activities were determined according to Ledeboer and Doern [32]. Other physiological and biochemical properties were tested using API 20NE (bioMérieux, Marcy l’Etoile, France). For these tests, cells were suspended in a solution of 0.85% of NaCl. Results were recorded after 48 h incubation at 30°C.

### Genetic identification

#### DNA extraction

Sterivex filters were thawed, and DNA was extracted following a modification of the method of Weon *et al*. and Maarit Niemi *et al*. [29,33]. Filters were filled with 1.8 ml sodium chloride-tris-ethylene-diamine-tetraacetic acid (EDTA) buffer (10 mM tris-hydrochloride [pH 8.0], 1 mM EDTA, 10 mM NaCl) containing fresh lysozyme (5 mg/mL) and sealed with a syringe and cap for a 1 h incubation at 37°C. A 3 μl aliquot of proteinase K (20 mg/mL) and 100 μl of 10% sodium dodecyl sulfate solution were added to the filter and incubated for 2 h at 60°C. Then 100 μl of NaCl (5M) was added to the tube, and 80 μl of cethyletrimethylammonium bromide was added and incubated for 10 min at 65°C. After that 600 μl of chloroform: isoamyle alcohol (24:1) was added to the tube and centrifuged at 1500 ×*g* for 5 min at 4°C. The clear part was transferred to a clean tube and equal volume of phenol: chloroform:isoamyle alcohol (25:24:1) was added to the tube.

The sample was centrifuged at 10,000 ×*g* for 5 min, and the supernatant was transferred to a clean tube. The DNA was precipitated by the addition of 300 μl 70% ethanol. By centrifugation at 14,500 ×*g* for 30 min, and the precipitated DNA was collected. After discarding the supernatant, the DNA was re-suspended in 400 μl tris-EDTA buffer and incubated at 37°C for 1 h.

#### Polymerase chain reaction (PCR)

The 16S rRNA was amplified using universal bacterial primers 27F and 1492R, according to Neefs *et al.*, 1990. PCR amplification was performed with 30 thermal cycles programmed as follows; denaturation for 1 min at 94°C, annealing for 2 min, 30 s at 55°C, an extension for 2 min, 30 s at 72°C, and with a final elongation step of 5 min at 72°C. The molecular size of PCR amplicon was determined by agarose electrophoresis. The PCR products (amplicon) were purified using Q1Aprep Spin Miniprep Kits prior to sequencing [34].

The 16S rRNA sequences were aligned using CLUSTAL W software Ver. 1.7 [35]. Kimura's two-parameter model [36] was applied for the calculations of evolutionary distance. A phylogenetic tree was constructed using the neighbor-joining method in MEGA 6 software [37]. Gb 03 sequences were deposited in NCBI GenBank with the accession numbers; *Loktanella* sp. strain UKMGb03A (KU199217), *Loktanella* sp. strain UKMGb03B (KU199218) and *Loktanella* sp. strain UKMGb03C (KU199219).

Fatty acid extraction was carried out by method described by Desbois and Smith [38]. The cellular fatty acid profiles of strain Gb-03 consisted of straight-chain saturated and unsaturated components with small amounts of hydroxyl.

Fatty acids; these profiles were almost similar to those of other members of the genus *Loktanella* [24,26,27,29,30]. The fatty acid profiles of strain Gb-03 is shown in Table-1.

**Table-1:** Cellular fatty acid content of strain Gb03.

Fatty acid	Gb03
Saturated	
C_16:0_	3.2
C_18L0_	4.2
Unsaturated	
C_16_	4.7
C_18_	3.8
C_20_	6.1
Hydroxy	
C_10_	0.7
C_15_	0.7

#### Algicidal activity

In this experiment, *Alxandrium* sp., *A. leei, A. affine*, *A. tamiyavanichi, A. tamarense, G. belizeanus*, and *Ostreopsis* were used to investigate the algicidal activity of the isolates. We used test flask, 10 ml of the *Dinoflagellates* culture at the mid-exponential growth phase (1.0 × 10^3^ 135 cell/mL) was added in the test flask, and 1 ml of the isolate culture was added to the test flask as well, to test the algicidal activity of Gb03 culture on *Dinoflagellate*. After 24 h incubation time the cells were observed using counting chamber (Sedgwick-Rafter cells [grid]).

## Results and Discussion

### Description of *Loktanella* sp. Gb-03

The cells were Gram-negative, aerobic and rod-shaped (0.6-0.86, 1.3-3.0 mm), non-motile. Colonies were circular, smooth, convex with an entire margin and pinkish in color. The isolates were observed to grow between 10 and 45°C and the optimum growth temperature was observed to be 30°C. The isolates were also found to grow in a range of pH between pH 6.0-12.0 and the optimum pH was found to be pH 7.0-8.0. The isolate grows on nutrient agar supplemented with 0.5-1.0% (w/v) NaCl, with optimum growth at 1%. While the isolates were able to grow in the presence of nutrient agar supplemented with NaCl, the bacteria were observed to be unable to grow in nutrient agar with no NaCl supplementation. The isolate was positive for catalase and oxidase activities, nitrate reduction, and gelatin liquefaction. But it was negative for glucose fermentation, arginine dihydrolase and indole production (API 20NE) (Table-2). The isolate does not grow in MacConkey broth and URE broth, but it grows in TSB (Difco) [24,27,29,30].

**Table-2:** Biochemical test on the isolates using API 20NE.

Colonies pigment	Pink-beige	Light orange	Pink	Pink	Light orange	Pink
API 20NE						
No3	+	−	+	+	−	+
TRP	+	−	−	+	−	−
GLU	+	−	−	−	−	−
ADH	−	−	−	−	−	−
URE	−	−	−	−	−	−
ESC	−	+	+	+	−	+
GEL	+	+	+	+	+	+
PNG	+	+	+	−	+	+
GLU	+	+	+	−	+	+
ARA	+	+	+	−	+	+
MNE	+	+	+	−	+	+
MAN	+	+	+	+	+	+
NAG	−	−	−	+	−	+
MAL	+	−	+	+	−	−
GNT	−	−	+	+	−	−
CAP	+	−	−	−	−	−
ADI	+	−	−	−	−	−
MLT	−	−	+	−	+	+
CIT	−	−	−	−	−	−
PAC	−	−	−	−	−	−

+=Positive, −=Negative, API=Analytical profile index

**Figure-1:**
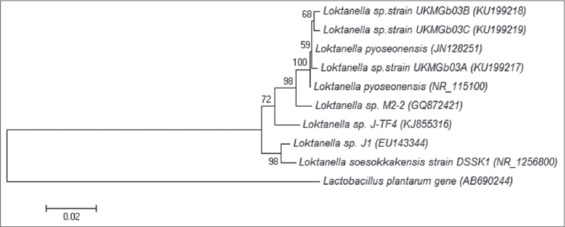
Phylogenetic three.

Following the amplification of the bacteria DNA with PCR, using 16S rRNA gene, the 1300 bp amplified DNA was sequenced, and the sequence of the 16S rRNA of Gb-03 strain was aligned in GenBank database with related available sequences. The nearest identity was those of *Loktanella* sp. (99%). Relationship between Gb-03 and genus *Loktanella* was very close (Figure-1) this is shown in the phylogenetic tree based on 16S rRNA sequence. Based on the biochemical analysis and phylogenetic tree, the isolate was identified as *Loktanella* sp.

### Emended description of the genus *Loktanella*

The cells are Gram-negative, strictly aerobic, moderately halotolerant, chemoheterotrophic, non-spore-forming and rod-shaped. Motility was observed to be variable among species and when observed, the cells were motile by means of flagella. The cells were cytochrome oxidase and catalase is positive. Colony colors were variable (white, pink, whitish pink, beige or light orange) depending on the species. The optimal temperature for growth was 25°C. The dominant fatty acid found on the isolate was C18: 1v7c while Q-10 was the major ubiquinone. The polar lipids were diphosphatidyl glycerol, phosphatidyl choline, and phosphatidyl glycerol. DNA G+C contents were 59.1-67.5 mol%. Phylogenetically, the genus belongs to the Rhodobacter group within the class Alphaproteobacteria. The species was identified to be *L. salsilacus* based on the ememded description of the genus [24]. Since it has been established that there are algicidal bacteria that kill algae and that these algicidal bacteria can be used as biological control agents [38], the isolation of an algicidal bacterium with proven algicidal activity from *Dinoflagellate* culture is a significant finding.

### Algicidal activity

Each of the six isolate (Gb-01, Gb-02, Gb-03, Gb-04, Gb-05 and Gb-06) showed the different percentage of algicidal activity on the different species of *Dinoflagellates* been tested in this study (Table-3). Among the six isolates, *Loktanella* sp. Gb-03 was observed to have the highest algicidal activity against a range of the tested bacteria following 24 h incubation, with the highest algicidal activity seen against *A. affine*, where the algicidal activity was observed to be as high as 95. In view of the fact that the effect of algicidal bacteria on microbial communities have been demonstrated to be clearly hazardous [38], the findings of this study could be of great value, especially when biotic control of HABs becomes necessary.

**Table-3:** Algicidal activity of isolates against some *Dinoflagellate* using NB as control.

Isolates	*A. tropicale*	*A. leei*	*A. affine*	*A. tamiyovon-ichi*	*A. tamarense*	*G. belizeanus*	*Ostreopsis*
Gb-01	50	40	50	40	50	50	50
Gb-02	40	40	30	40	40	50	40
Gb-03	70	50	95	70	90	90	60
Gb-04	30	30	30	50	70	70	50
Gb-05	70	30	40	50	60	70	50
Gb-06	80	60	50	50	40	50	40

*A. tropicale=Alexandrium tropicale, A. tamarense=Alexandrium tamarense, A. leei=Alxandrium leei, A. affine=Alxandrium affine, A. tamiyovon-ichi=Alexandrium tamiyovon-ichi, G. belizeanus=Gambierdiscus belizeanus*, NB=Nutrient broth

## Conclusion

The isolated marine bacteria in this study showed a powerful algicidal activity against wide range of *Dinoflagellates* species, indicating the potential of Malaysian marine as a good source of natural algicides, especially in the biotic control of HABs. This appears to be the first study to isolate and investigate marine algicidal bacteria and to investigate their algicidal effect against toxic *Dinoflagellates* in Malaysian water. The isolate retained algicidal stability at the optimum growth conditions, suggesting that the isolate is capable of being stable especially during the manufacturing process and in the applied area.

## Authors’ Contributions

AHB and AA designed the experiment and conducted the experimental work. AHB, GU, and AA were involved in scientific discussion and analysis of the data. GU and AA drafted and revised the manuscript. All authors read and approved the final manuscript.
